# Enlargements of the Liver.—VIII

**Published:** 1910-02-26

**Authors:** 


### Medicine.

### ENLARGEMENTS OF THE LIVER.—VIII.

### Diabetes Mellitus.

In addition to the hepatic changes in bronzed diabetes already discussed, it is not uncommon to find the liver enlarged and fatty in ordinary diabetes mellitus. The edge is a short distance below the costal margin, and the surface is usually smooth and not tender. Its occurrence with urine of a high specific gravity and containing a good deal of sugar would give the probable diagnosis.

### Phosphorus Poisoning.

In a typical case of phosphorus poisoning the liver is enlarged, very fatty, and extremely tender to pressure. Jaundice occurs early in some cases, but in others it may be delayed until the sixth or seventh day. The urine is diminished in total quantity, its specific gravity varies between 1,020 and 1,037, and its reaction is strongly acid. Albuminuria is common, but not invariably present. The total nitrogen in the urine is at first reduced to 2 to 5 grammes daily, but this condition is followed by a rise to 10 to 17 grammes per day. Notwithstanding the gross changes in the liver, urea still forms the chief nitrogenous constituent of the urine. The diagnosis is not easy unless there is a history of something containing phosphorus having been taken by the patient.

### Carcinoma of the Liver.

In this disease the liver may become enormously enlarged, even up to as much as a weight of 22 lb. Indeed, the enlargement from this cause may be greater than that from any other. The carcinoma may be primary or secondary. Secondary is very much more common than primary, the original seat of the growth being very variable—stomach, colon, rectum, gall-bladder, bile-duct, pancreas, breast, uterus, and so forth. In addition to being enormously enlarged, the organ is irregular in form, of stony hardness, nodular on the surface, and with an irregular and nodular edge. The nodules which project from the surface are usually depressed at the centre or umbilicated as the result of the breaking down and softening of their central parts, leading to a falling in of the projecting and therefore unsupported parts. A naked-eye section of the organ shows nodules of growth of varying sizes scattered throughout it.

Primary carcinoma of the liver may be nodular or diffuse. In the nodular variety there is usually one very large mass, the primary seat of the growth, with a number of smaller secondary nodules, so that the organ is enlarged, irregular, and bossy, just as it is in cases of secondary carcinoma. In the diffuse variety the liver may be uniformly enlarged; it is, however, very hard as a rule, resembling the irregular secondary carcinomatous liver in this respect. Primary diffuse carcinoma, it is said, is apt to be associated with cirrhosis, and even at a post-mortem examination it may be difficult to determine, without microscopical examination, whether the condition is due to cirrhosis or to carcinoma or to both.

The physical signs in the secondary and in the primary nodular forms are: Distension of the abdomen, especially in the epigastric and right hypochondriac regions, the local prominence depending entirely on which part of the liver is most affected; enlargement of the superficial abdominal veins; flattening and often fixation of the umbilicus; when emaciation is well marked and the abdominal wall is thinned the hepatic nodules become plainly evident beneath the skin, and they may be seen to move downwards behind the abdominal wall when the patient inspires deeply; when the enlargement is considerable, the edge of the liver, irregular, coarsely nodular, and very hard, may be felt at the level of the umbilicus in the right nipple line, or, in advanced cases, even down to the level of the iliac crest; it may be traced across the abdomen below the umbilicus into the left hypochondriac region; the hepatic surface is extremely hard and irregular from projecting nodules of growth of varying sizes, some of them presenting central depressions or umbilication; if watched from day to day the liver may be found to increase in size gradually but progressively; the enlargement usually takes place in a downward direction so that the upper limit of dulness remains the same; the weight of the organ may sometimes lead to downward dislocation, however, whilst in the case of a primary nodular carcinoma developing in the upper part of the right lobe the diaphragm may be pushed upwards, the base of the right lung compressed, and the outline of the upper limit of hepatic dulness being as much convex or dome-shaped as it is with sub-diaphragmatic abscess, tropical hepatic abscess, or hydatid cyst of the liver.

In diffuse primary carcinoma the liver may be uniformly enlarged, the edge hard, sharp, and well defined, the surface very hard, but smooth and often tender.

The chief symptoms are: Severe dull or sharp pain in the right hypochondrium and right shoulder, gradual and progressive emaciation and loss of strength, loss of appetite, nausea, and vomiting, cachexia, jaundice, which may be intense if the common bile-duct is involved, ascites if the portal vein or its branches are compressed, pyrexia, anæmia, and slight œdema of the legs.

It is often impossible to say whether growth of the liver is primary or secondary unless definite evidence of a primary growth is found. In many cases in which there is no evidence from symptoms or physical signs of a primary growth it would be rash to diagnose primary carcinoma of the liver, for the original seat of the neoplasm is frequently not apparent without a post-mortem examination.

It should be noted that in every obscure case of the kind a rectal examination should invariably be made; not infrequently the only symptoms of a rectal carcinoma are those due to the secondary deposits in the liver.

Sarcoma.

Sarcoma of the liver may be primary or secondary, though the primary is exceedingly rare. In either case it usually takes the form of nodules of new growth, which vary much in size, which are not as a rule strictly circumscribed, and which project from the surface of the organ. Melanotic sarcoma is one of the least rare varieties of this rather uncommon malady, and it is as a rule secondary to similar growth in an eye or in the skin. The liver may become enormously enlarged. A sarcomatous tumour of the liver may increase in size very rapidly, and it is often exceedingly vascular, so that, in addition to the presence of a nodular enlargement of the liver, it may be possible to detect both pulsation and a bruit in the mass. If there are numerous nodules of what appear to be secondary growth in the skin sarcoma is more likely than carcinoma. In cases of melanotic sarcoma the urine, after standing exposed to the air, may darken and be found to contain melanin.

The most important symptoms are anæmia, weakness, rapid emaciation, pain in the hepatic region, and possibly ascites and jaundice. If the primary growth has been detected, the diagnosis is easy; otherwise it may be very difficult. If the primary growth is not known, and yet the diagnosis has been narrowed down so as to lie between secondary carcinoma and secondary sarcoma of the liver, the great rapidity of the development of the nodular enlargement would be in favour of sarcoma.

(*To be continued.*)

